# A degradation feature extraction technique based on static divided symbol sequence entropy

**DOI:** 10.1038/s41598-023-50575-6

**Published:** 2024-01-03

**Authors:** Chunxia Gu, Juan Bi, Bing Wang

**Affiliations:** 1 School of Economics & Management, Shanghai Maritime University, Shanghai, China; 2 School of Tourism and Geography Science, Qingdao University, Qingdao, China; 3 Logistics Engineering College, Shanghai Maritime University, Shanghai, China

**Keywords:** Engineering, Mechanical engineering

## Abstract

Due to the doping of considerable noise and impact components in vibration signals of quay crane gearboxes, some traditional methods have difficulty uncovering degradation patterns. To accurately extract degradation features from vibration monitoring signals, a degradation feature extraction technique based on static divided symbol sequence entropy is proposed. Based on the basic scale entropy technique, considering the uniformity of the symbolization standard, the technique takes the root mean square of the health condition signal as the basis and incorporates the scale coefficient to establish a uniform basic scale. Simultaneously, the symbol set is expanded to enhance the information content and the ability of the approach to characterize the complexity levels of signals in large-value regions. Therefore, the proposed static divided symbol sequence entropy technique can accurately and flexibly characterize performance degradations. The logistic chaotic sequence and the lifetime signal of hoisting mechanism gearbox are separately used for analysis. It shows that the proposed technique can characterize the complexity of the nonlinear time series and sensitively describe the performance degradation exhibited by hoisting mechanism gearbox. This technique is computationally fast, and it can be implemented as a foundation for developing new methods for evaluating the health conditions of quay crane gearboxes.

## Introduction

Quay cranes are widely used large-scale port hoisting machinery that mainly complete loading and unloading functions for port containers. The hoisting reduction box is a key transmission component of the hoisting mechanism, and its running condition directly affects the safety and reliability of the system. A quay crane usually works in harsh natural environments and special working conditions; therefore, the vibration monitoring signals of the hoisting reduction box present nonlinear, non-stationary and non-periodic characteristics. At the same time, the vibration signals are also mixed with many noise and shock components under working conditions, which increase the difficulty of vibration signal analysis^1^. In addition, a quay crane has a long lifetime and a harsh working environment, so it is difficult to monitor and verify the technique’s effectiveness online. Therefore, determining how to extract the degradation features that characterize the operating conditions of cranes from complex and special monitoring signals is of great research significance for accurately assessing the health conditions of quay cranes and implementing condition based maintenance (CBM)^2^.

As the basis of health condition evaluation, degradation feature extraction is used to quantitatively determine the performance degradation principle contained in a signal^3^. In view of the nonlinear, non-stationary and non-periodic characteristics of vibration monitoring signals, in recent years, signal analysis methods based on complexity theories, including information entropy, fractal dimensions and chaos, have been widely used in degradation feature extraction methods for rotating machinery such as rolling bearings and gears. Some techniques, such as fuzzy entropy^4^, permutation entropy^5^, dispersion entropy^6^, amplitude spectrum entropy^7,8^, and fractal dimensions^9,10^, have been proposed. For example, a degradation feature extraction method based upon morphological undecorated wavelet decomposition fusion (MUWDF) and DCT high-order singular entropy is proposed in the literature^11^. A hybrid domain feature extraction method based on the distance evaluation technique (DET) was proposed in^12^. In^13^, a developed logarithmic normal distribution-based variational autoencoder algorithm that can ensure that the final feature extraction results follow the log-normal prior hypothesis was proposed. A method based upon the modified composite spectrum and relative entropy was proposed for degradation feature extraction in^14^.

Because of the difficulty of collecting lifetime vibration monitoring signals, accelerated test data from IMS (Intelligent Maintenance Systems, IMS)^15^ and IEEE PHM (Prognostic and Health Management, PHM) 2012^16^ are usually introduced for verification purposes. Compared with the accelerated lifetime signals described above, quay crane vibration signals are irregular and doped with more noise and shock components, and some effective methods verified on accelerated test data have been proven to not work when processing vibration signals from quay cranes.

Symbol dynamics is a theoretical signal symbolization method that has been successfully applied in the fields of network security^17^, bio-medicine^18^, and mechanical engineering^19^. This method is used by digitizing the original time series and extracting its main trend. At the same time, the noise components can be reduced, thereby greatly improving the effect and calculation efficiency of the method. The combination of symbol dynamics and complexity theory can be used to quantitatively and effectively extract the complexity and characterize complex nonlinear systems.

Based on the above analysis, to effectively extract the degradation features of a quay crane hoisting gearbox, a degradation feature extraction method based on static divided symbol sequence entropy is proposed by combining symbol dynamics theory and information entropy on the basis of the existing basic scale entropy. An analysis and verification are carried out with the logistic sequence and lifetime dataset derived from a gearbox, respectively.

The contribution of this proposed technique can be illustrated in the following two aspects. First, considering the uniformity of the symbolization standard, the technique takes the root mean square of the health condition signal as the basis and incorporates the scale coefficient to establish a uniform basic scale. Second, the symbol set is expanded to enhance the information content and the ability of the approach to characterize the complexity levels of signals in large-value regions.

The paper is organized as follows. In “Theory of basic scale entropy” section, basic scale entropy is briefly introduced. In “Proposed static divided symbol sequence entropy method” section, the new technique named static divided symbol sequence entropy is proposed. A simulated analysis of a logistic sequence is provided in “Simulated analysis of a logistic sequence” section. In “Instance data analysis” section, the proposed method is verified, and the results are discussed. Finally, the conclusion of this paper is given in “Conclusion” section.

## Theory of basic scale entropy

The entropy-based method is an effective method for degradation feature extraction. Among the entropy-based methods, basic scale entropy (BSE) is a typical feature analysis method based on static symbol division. It is mainly used for processing ECG (electrocardiogram) signals in the medical field^20^. In recent years, it has also been employed in some preliminary applications in the field of mechanical equipment fault diagnosis^21^. The principle of the BSE method is as follows:

It is supposed that *u* is a one-dimensional time series with a length of *N*. The sequence is transformed in the phase space as follows:

1 $$X\left(i\right)=\left[u\left(i\right),u\left(i+L\right),\ldots ,u\left(i+\left(m-1\right)L\right)\right]$$

where *m* is the transformation dimension and *L* is the delay factor, which requires that *i* + (*m* − 1) *L* ≤ N. Generally, *L* = 1, and the structure of matrix *X* is (*N* − *m* + 1) × *m*. Later, each m-dimensional vector is symbolized and converted to a sequence S with m-dimensional vector symbols.

2 $${S}_{i}\left({X}_{i}\right)=\left\{s\left(i\right),s\left(i+L\right),\dots ,s\left(i+\left(m-1\right)L\right)\right\}$$

In the above formula, *s* ∈ A:A = {0,1,2,3}, and the principle of symbolization is as follows:

3 $${S}_{i}({X}_{i})=\left\{\begin{array}{l}0:\overline{u}<{u}_{i+k}\le \overline{u}+a\times BS\\ 1:{u}_{i+k}>\overline{u}+a\times BS\\ 2:\overline{u}-a\times BS<{u}_{i+k}\le \overline{u}\\ 3:{u}_{i+k}\le \overline{u}-a\times BS\end{array}\right.$$

where *ū* and *BS* represent the mean and basic scale of the *i*th m-dimensional vector, respectively, and *BS* is defined as follows:

4 $$BS(i)=\sqrt{\frac{\sum_{j=1}^{m-1}{\left[u(i+j)-u(i+j-1)\right]}^{2}}{m-1}}$$

In the above formula, the argument *a* is used as the basic scale parameter, which needs to be properly selected in practical applications. This method uses *a* × *BS* as the symbolization standard.

The symbol pattern distribution probability *P* in sequence S is calculated. The total number of patterns of the four symbols is *π* = 4^*m*^, and the probability of the symbol pattern in S is calculated as follows:

5 $$p(\pi )=\frac{Num\{t|({u}_{t},{{u}_{t+}}_{1},...,{u}_{t+m-1}) hastype \pi \}}{N-m+1}$$

Among them, 1 ≤ *t* ≤ *N* − *m* − 1, and Num represents the number of patterns.

Finally, according to the basic definition of information entropy, the BSE is calculated as follows:

6 $$H(m)=-\sum p(\pi ){\mathit{log}}_{2}p(\pi )$$

According to the principle of BSE, this method can be used to describe the fluctuation pattern of the input sequence. The larger the resulting value is, the more complex the fluctuation pattern, and vice versa.

## Proposed static divided symbol sequence entropy method

According to the discussion on the basic theory of BSE in “Proposed static divided symbol sequence entropy method” section, it is clear that the BSE method takes *a* × *BS* as the symbolization standard to quantitatively measure the fluctuation pattern of a sequence, and the basic scale *BS* of each series must be calculated according to Formula (4). This yields two shortcomings. First, the inconsistency of the basic scale represents a difference in the symbolization standard, and it is difficult to uniformly measure sequence complexity changes. Second, the length of the time sequence affects the calculation speed, and the value of parameter *a* affects the symbolization standard.

Based on the above considerations, a feature extraction method called static divided symbol sequence entropy (DSSE) is proposed. Its working principle is as follows.

Step 1: *u* is assumed to be a one-dimensional time series with a length of *N*; and *u* is converted into an m-dimensional vector *X*.
  7 $$X\left(i\right)=\left[u\left(i\right),u\left(i+L\right),\dots ,u\left(i+\left(m-1\right)L\right)\right]$$
Step 2: Each *m*-dimensional vector is symbolized in turn and converted into an *m*-dimensional vector symbol sequence S and the number of symbols is defined as A .
  8 $${S}_{i}\left({X}_{i}\right)=\left\{s\left(i\right),s\left(i+L\right),\dots ,s\left(i+\left(m-1\right)L\right)\right\}$$
  where *s* ∈ A:A = {0,1,…,*K*}. The conversion principle is as follows:
  9 $${S}_{i}({X}_{i})=\left\{\begin{array}{l}1,\left|u(i)\right|\le B{S}_{0}\\ 2,B{S}_{0}<\left|u(i)\right|\le 2\times B{S}_{0}\\ \cdots \\ j,(j-1)\times B{S}_{0}<\left|u(i)\right|\le j\times B{S}_{0}\\ \cdots \\ K,\left|u(i)\right|>K\times B{S}_{0}\end{array}\right.$$
  Among them, *K* denotes the number of symbols, and the symbol set is A = {1,2,3,4,5} when *K* = 5. *BS*_*0*_ is defined as the basic symbolization scale, compared with *BS*_*0*_ in Formula (4). To evaluate the variation under the same standard, this parameter is set to a fixed value. In this paper, this parameter is set as the product of the root mean square value of the first group of health condition signals and the coefficient *a* as follows. Under the same standard *BS*_*0*_, the signal could be symbolized from a continuous amplitude to a numerical symbolization as shown in Formula (9).
  10 $$B{S}_{0}=a*rms\left({u}_{1}\right), \quad 0<a<2$$
  In this method, *K* represents the size of the symbol set, which is the number of symbolized regions. The base scale representation signifies the size of the symbolized area.
Step 3: The symbol pattern distribution probability *P* in S is counted. The arrangement pattern of the *K* symbols is *π* = *K*^*m*^, and the calculation method is the same as that in Formula (5).
Step 4: Following the basic definition of information entropy and BSE in Formula (6), the entropy *DSSE* of the static divided symbol sequence entropy can be calculated as follows:
  11 $${H}_{0}(m)=-\sum p(\pi ){\mathit{log}}_{2}p(\pi )$$

To compare and analyze the influence of parameter selection on the results, the sequence entropy calculation is not normalized in this paper.

## Simulated analysis of a logistic sequence

**Figure 1:**
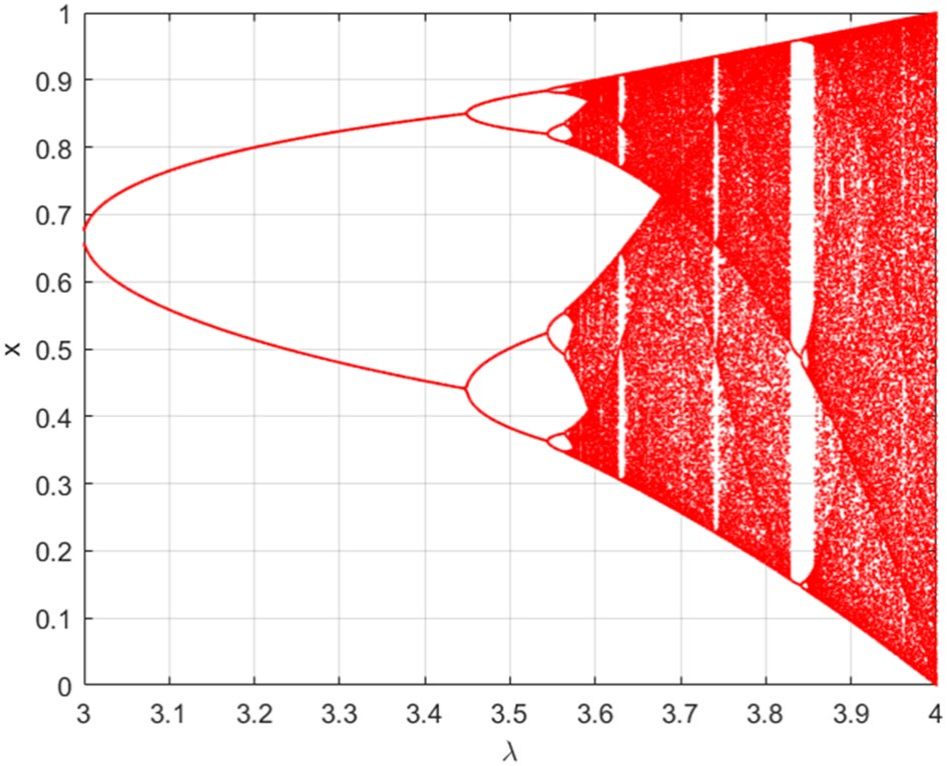
Logistic model bifurcation diagram. The bifurcation diagram of logistic model is shown in this figure. For this model, the initial value is set as 0.4, and the sequence length is set as 2000. When* λ* is greater than 3.446, the logistic model begins to oscillate, and when *λ* is approximately equal to 3.567, it begins to enter a chaotic state.

To evaluate the effectiveness of the proposed DSSE technique, a logistic model is proposed to verify its ability to indicate complexity. The logistic model, also known as the ‘Pest model’^22^, is a typical second-order recursive polynomial* x*_t_ + 1 = *λ*⋅*x*_t_(1 − *x*_t_), which is mainly used in nonlinear dynamics and chaos analysis. The bifurcation diagram of the logistic model (the initial value is 0.4, and the sequence length is 2000) is shown in Fig. 1. When *λ* is greater than 3.446, the logistic model begins to oscillate, and when *λ* is approximately equal to 3.567, it begins to enter a chaotic state. As *λ* increases, the complexity of the sequence also increases, but it also includes no chaotic sub-sequences, such as the islands of the stability phase between *λ* = 3.829 and 3.86 when the sequence returns from the chaotic state to the initial oscillating state.

**Figure 2:**
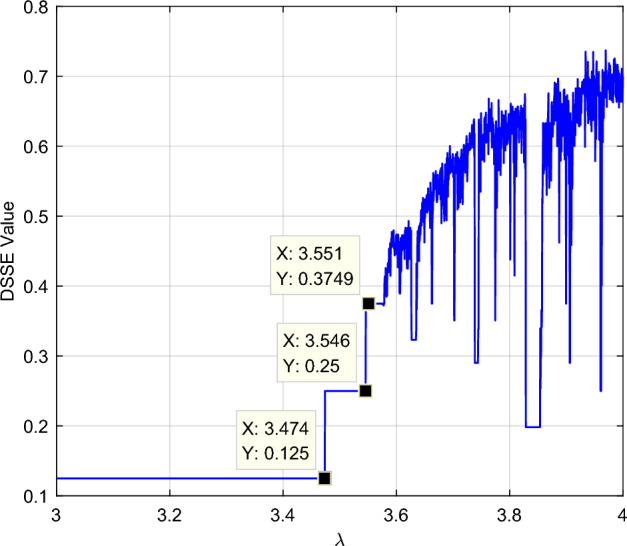
DSSE degradation feature sequence of the logistic model. The DSSE degradation feature curve is shown in this figure. The DSSE curve effectively characterizes the process of model complexity from low to high, and it is simultaneously sensitive to the initial oscillation at *λ* = 3.446 and the islands of the stability phase.

The logistic model is processed by the proposed static divided symbol sequence entropy approach. The selection of parameter a has a certain impact on the calculation of symbolization standards, and multiple experiments have shown that parameter a is effective when selected within the interval [0,1]. Therefore, in simulation analysis, parameter a is set to* a* = 0.2, and the effective value of the first group of sequences is 0.666, so the basic scale *BS*_*0*_ = *a* * 0.666. The number of symbols is set as *K* = 6, and the DSSE degradation feature curve is shown in Fig. 2. It is obvious that the DSSE curve effectively characterizes the process of model complexity from low to high, and it is simultaneously sensitive to the initial oscillation at *λ* = 3.446 and the islands of the stability phase, indicating that this method can effectively characterize the complexity of the input nonlinear time series.

**Figure 3:**
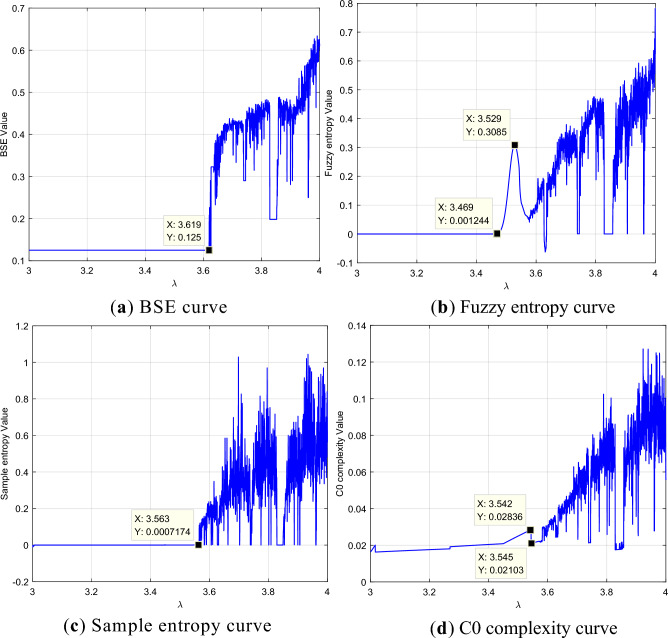
Complexity-based feature degradation curves of the logistic model. (**a**) The degradation curves based on BSE is shown in this figure, and the curve shows a overall upward trend during the evolution process, however, the initial oscillation of the sequence can not be reflected. (**b**) The degradation curves based on Fuzzy entropy is shown in this figure, and the curve shows a overall upward trend during the evolution process, however, the initial oscillation of the sequence can not be reflected. (**c**) The degradation curves based on Sample entropy is shown in this figure, and the curve shows a overall upward trend during the evolution process. (**d**) The degradation curves based on C0 complexity is shown in this figure, and the curve shows a overall upward trend during the evolution process.

A horizontal comparison is made using typical complexity calculation methods, including BSE^22^, fuzzy entropy^23^, sample entropy^24^, and C0 complexity^25^. The degradation curves of the four methods are shown in Fig. 3. Regarding the four methods, the overall upward trend is reflected well during the evolution process, but the BSE and sample entropy curves fail to reflect the initial oscillation of the sequence, and the complexity produced by the fuzzy entropy method in the initial oscillation process appears ‘distorted’.

The parameters and computational performance of the above methods are shown in Table 1. The operating environment contains an Intel Xeon^®^ CPU E5-2450 L, 16.0 GB of memory, and MATLAB 2014a. In terms of the operation time, C0 complexity is the fastest, while both fuzzy entropy and sample entropy are slower. The main reason for this is that C0 complexity is a typical structural complexity method and only involves a Fourier transform and statistical analysis, so it has a calculation process. Fuzzy entropy and approximate entropy are two typical behavioral complexities. Due to the phase space transformation involved in the calculation process, the number of calculations is large, and the calculation time is long. A phase space transformation is also included in the calculation process of the DSSE technique, but the number of calculations is greatly reduced due to the symbolization process. Therefore, the operation speed and structural complexity are almost the same order of magnitude. Therefore, the proposed static divided symbol sequence entropy technique can reflect the change trend of signal complexity, its operation is accurate, and its calculation process is fast. It is suitable for extracting the degradation features of mechanical equipment.

**Table 1:** Settings and calculation times of typical complexity degradation feature extraction methods.

Methods	Parameter settings	Time (s)
Basic scale entropy	*m* = 4, *a* = 0.5	0.8931
Static divided symbol sequence entropy	*BS*_*0*_ = 0.2 * rms(*u*_*1*_); *u*_*1*_ is the first group signal	0.8433
Fuzzy entropy	*m* = 4, *r* = 0.2 * std(s), n = 10	32.5798
Sample entropy	*m* = 4, *r* = 0.2 * std(s)	10.9290
C0 complexity	*a* = 0.8	0.3937

## Instance data analysis

### Lifetime vibration loading spectrum of a quay crane gearbox

**Figure 4:**
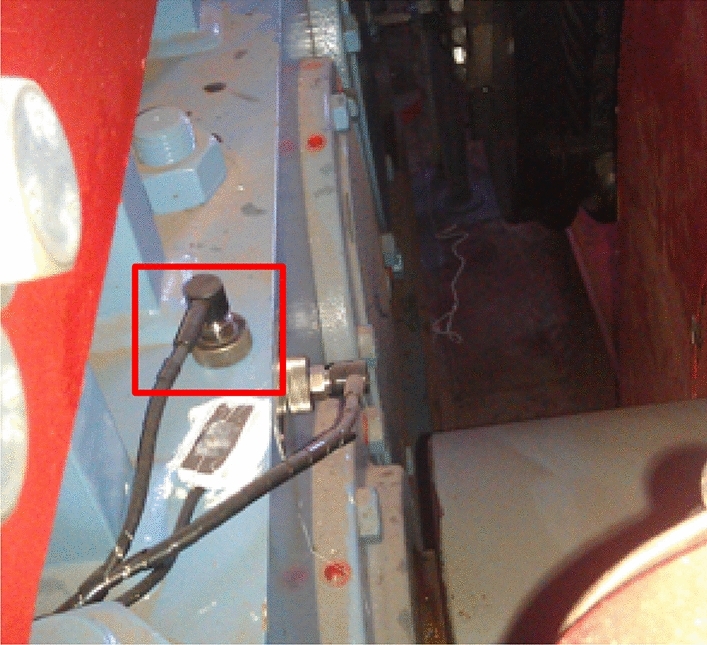
Vibration sensors. Reproduced from Sun et al.^26^. The full lifetime data are derived from the vertical vibration sensor of the high-speed input shaft of the hoisting gearbox is shown in this figure. The signal sampling frequency was 24 kHz, the sampling time was 1 s, and the sampling interval was set as 10 s.

**Figure 5:**
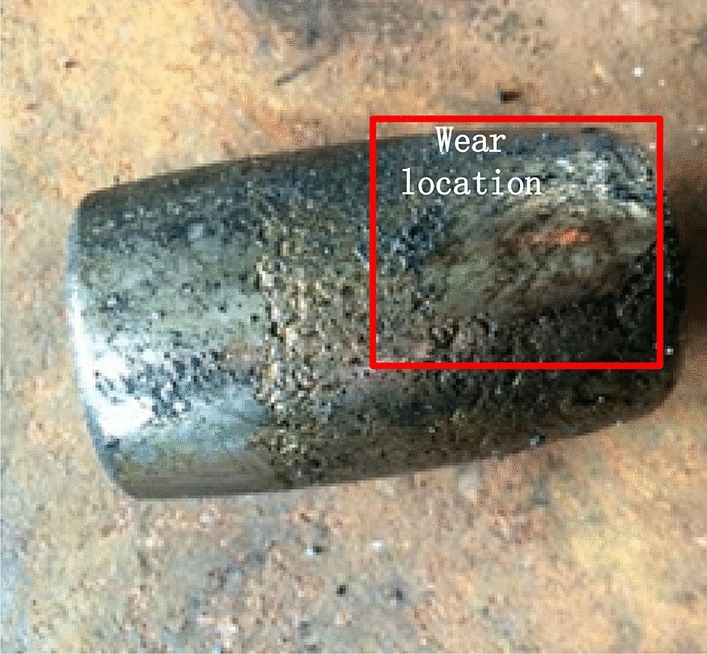
Realistic diagram of a roller bearing fault. Reproduced from Sun et al.^26^. When some faults occurred on the gearbox, the fault location was found at the high-speed input shaft roller bearing of the gearbox, and the failure mode was roller wear, the figure is shown in this figure.

The study object is the gearbox of the quay crane hoisting mechanism of a container terminal in Shanghai Port, and the signal monitoring platform is the network crane condition monitoring and assessment system (Net-CMAS). This system monitors vibration, temperature and stress signals online. The full lifetime data are derived from the vertical vibration sensor of the high-speed input shaft of the hoisting gearbox shown in Fig. 4. The signal sampling frequency was 24 kHz, the sampling time was 1 s, and the sampling interval was set as 10 s. Every time a data point was collected, the system automatically calculated the effective value and stored it as a sample point of the vibration loading spectrum sequence. After nearly 7 years and 8 months of online crane monitoring, some faults occurred at the hoisting gearbox. After shutting down for maintenance, it was found that the fault location was the high-speed input shaft roller bearing of the gearbox, and the failure mode was roller wear, as shown in Fig. 5.

**Figure 6:**
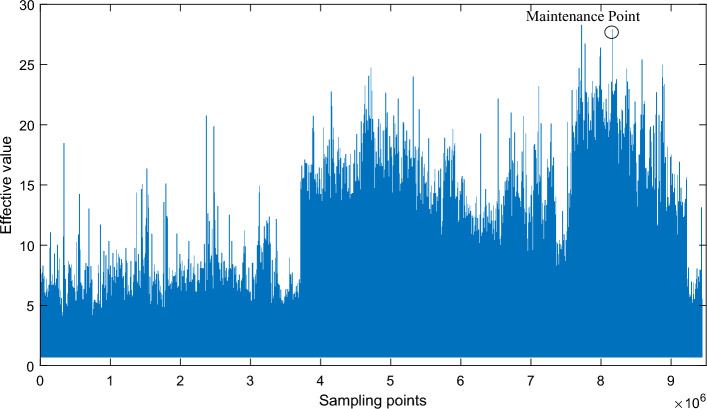
Time waves of the whole loading spectrum. After collecting the whole lifetime vibration loading spectrum of the hoisting gearbox from health to failure, the time-domain wave and the maintenance point is shown in this figure.

During the monitoring period, the Net-CMAS system collected the whole lifetime vibration loading spectrum of the hoisting gearbox from health to failure (the effective value sequence of each sampled signal). The time-domain wave and the maintenance point are shown in Fig. 6.

To facilitate the analysis, the massive loading spectrum is stored in the form of a 4608 * 2048 matrix, and each row represents the loading spectrum sequence of approximately 1 day, including a total of 4608 rows of data.

### Influence analysis of the parameters

**Figure 7:**
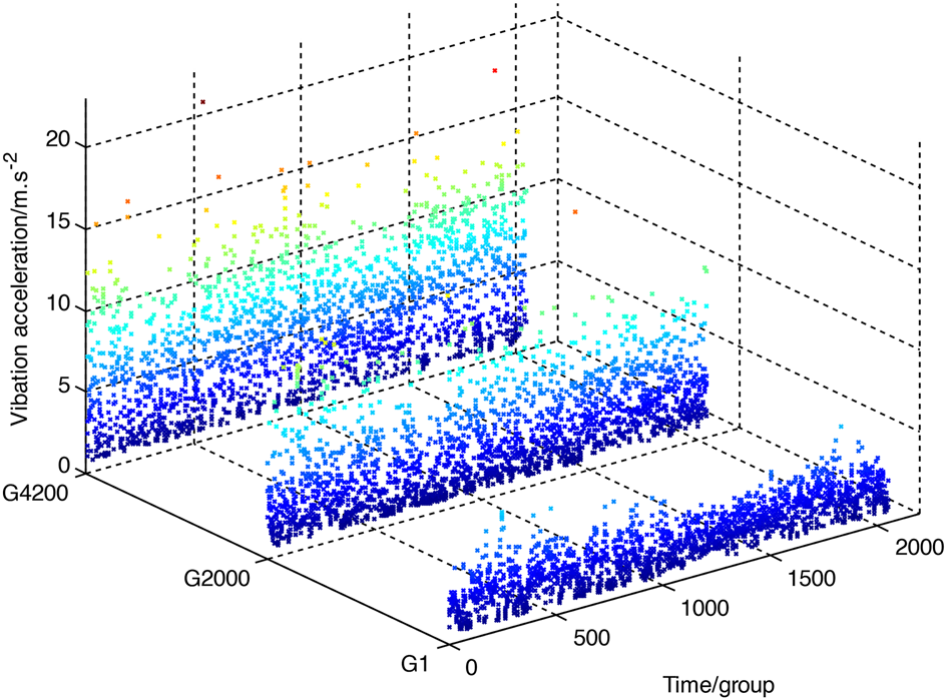
Time waves of three groups of loading spectra. Three typical vibration loading spectrum is shown in this figure, including G1,G2000 and G4200, representing different stage of the gearbox during the whole lifetime monitoring.

Three groups of vibration loading spectra are taken as examples to analyze the influence of the model parameters. The samples include the 1st, 2000th, and 4200th groups and are named G1, G2000, and G4200_,_ respectively. The loading spectrum time series is shown in Fig. 7. The larger the loading spectrum group is, the larger the proportion of the large amplitude component. Additionally, since the loading spectra store the root mean square value of each group signal, they are all positive values.

**Figure 8:**
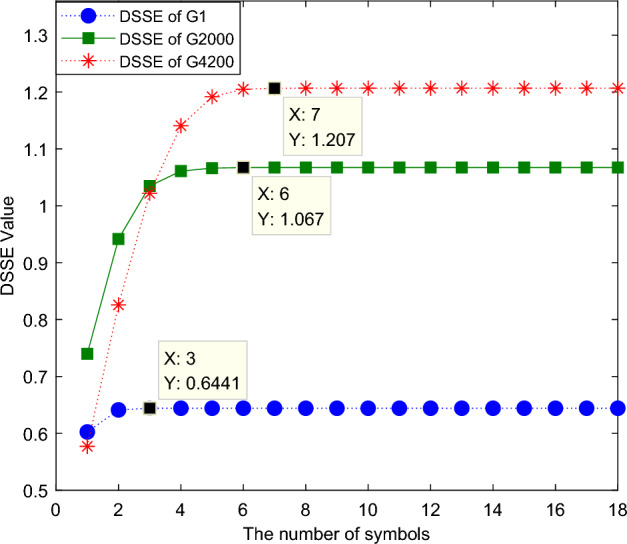
Correlation curve between the DSSE value and the number of symbols. The parameters are set as *m* = 4 and *a* = 0.6, and the basic scale of divided symbolization is set as *BS*_*0*_ = a * 0.666 = 1.1270. The number of symbols is set from 3 to 18. Under each number of symbols, three groups of DSSE value change curves are calculated and shown in this figure.

The number of symbols *K* can be used to determine the size of the symbol set. The parameters are set as *m* = 4 and *a* = 0.6, and the basic scale of divided symbolization is set as *BS*_*0*_ = a * 0.666 = 1.1270. The number of symbols is set from 3 to 18. Under each number of symbols, three groups of DSSE value change curves are calculated, and the results are shown in Fig. 8. As the number of symbols increases, the DSSE value gradually increases, but it remains stable after a certain number of symbols. The main reason for this finding is that the number of symbols directly determines the number of symbol sequence patterns (*K*^*m*^). The initial number of symbols is small, and the pattern distribution of the symbol sequence is uneven. As the number of symbols increases, the pattern of the symbol sequence tends to become more uniform; therefore, the value gradually increases. As the value of *K* increases, when a newly added symbol standard is too high to symbolize the amplitude of the corresponding sequence, the DSSE value no longer increases. At the same time, it can be seen that the smaller the group is, the smaller the number of symbols when the DSSE value is stable, which is consistent with the above analysis; that is, the number of symbols can gradually cause the information expression ability of the symbol sequence to increase for high-amplitude signals and the complexity resolution of high-amplitude signal regions to improve. Considering that the three sets of curves are stable when K > 7, when using the DSSE technique, *K* is set as 8 in this paper.

**Figure 9:**
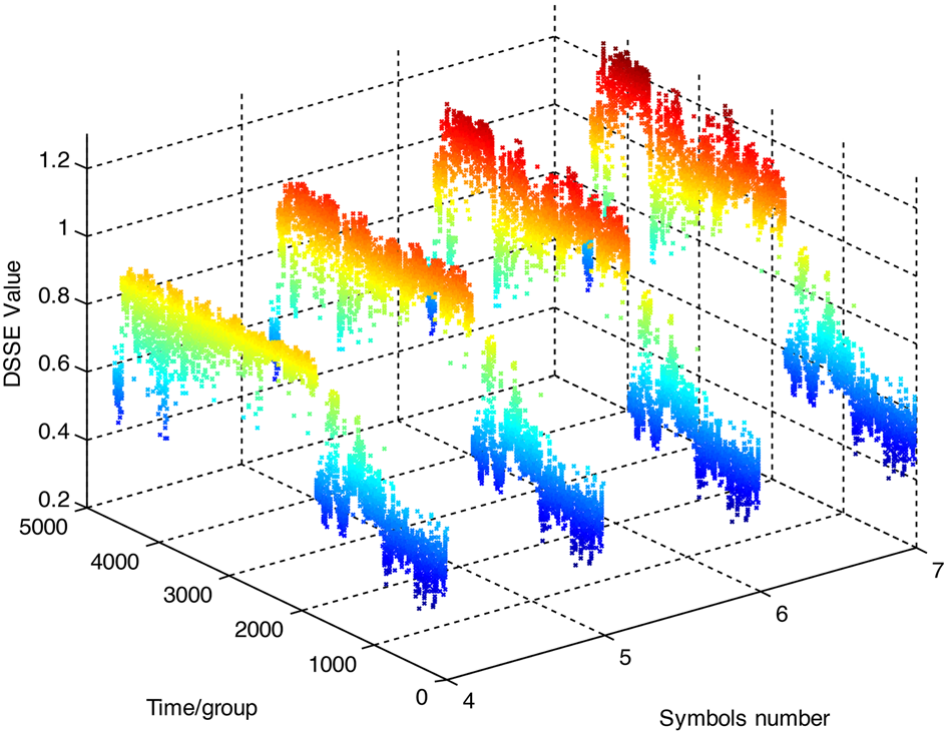
DSSE degradation curves produced under different numbers of symbols. The DSSE degradation characteristic curves produced when the number of symbols is set as 4, 5, 6, and 7 is shown in this figure. The greater the number of symbols, the larger the overall value is.

The DSSE degradation characteristic curves produced when the number of symbols is set as 4, 5, 6, and 7 are shown in Fig. 9. It is clear from the comparison that the greater the number of symbols, the larger the overall value is. With the increase in the number of symbols, the DSSE value in the low-amplitude area gradually becomes stable, and the value of the high-amplitude area gradually increases, improving the ability of the method to distinguish this area. This is consistent with the analytical conclusion derived from the above figure.

**Figure 10:**
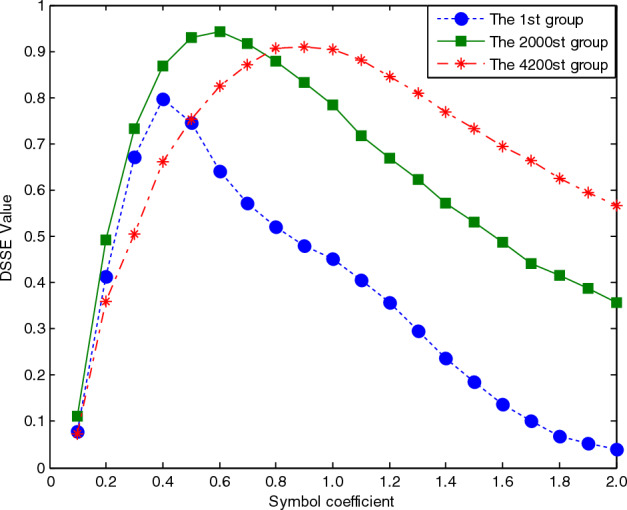
Correlation curve between the DSSE value and the basic scale coefficient. The parameters are set as *m* = 4 and *K* = 8, the basic scale of the divided symbolization is set as *BS*_*0*_ = a * 0.666, and the value of a is set from 0.1 to 2. The DSSE curves produced for the three groups under different scale coefficients are shown in this figure.

The value of the basic scale coefficient *a* can be used to determine the size of the symbol area. The parameters are set as *m* = 4 and *K* = 8, the basic scale of the divided symbolization is set as *BS*_*0*_ = *a* * 0.666, and the value of *a* is set from 0.1 to 2. The DSSE curves produced for the three groups under different scale coefficients are shown in Fig. 10. It is clear that with the increase in the scale coefficient, the symbolized area also increases, and the DSSE value gradually increases, but the value gradually decreases after a certain level. The main reason for this phenomenon is that the larger the scale factor is, the wider the symbolized area. For the same group, when the number of symbols is unchanged, with the increase in the coefficient, the symbol sequence pattern can become more uneven, and the symbol sequence coverage increases. Thus, the entropy value of the sequence increases. After reaching the maximum value, the symbolized area continues to increase. At this time, the coverage pattern tends to be uneven, and the high-amplitude symbol sequence pattern increases, thereby reducing the DSSE value.

**Figure 11:**
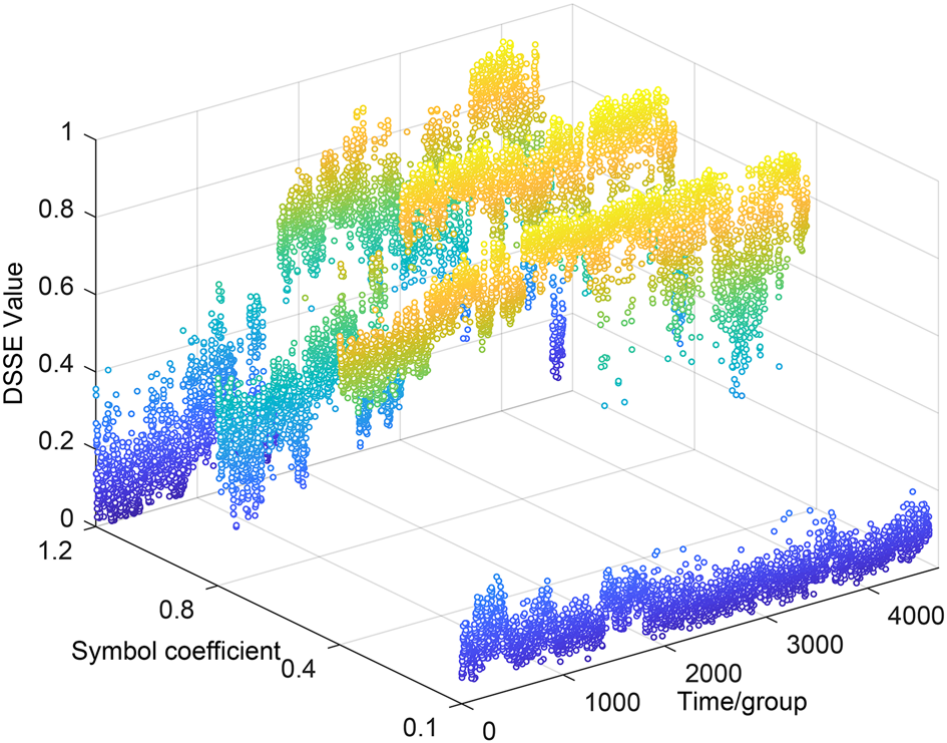
DSSE degradation curves produced under different basic scale coefficients. The DSSE degradation characteristic curves produced when the basic scale coefficient is set to 0.1, 0.4, 0.8, and 1.2 is shown in this figure. When the coefficient is small, the overall degradation curve is distorted, which indicates that the selected parameter is unreasonable. As the parameter value increases, the overall resolution gradually increases and becomes stable.

At the same time, the smaller the group is, the smaller the value obtained when reaching the DSSE maximum. Therefore, the basic scale factor can be used to control the accuracy of the symbol sequence's ability to express signal information. When the value is greater than 0.8, the DSSE curves of the three groups are basically stable. Therefore, this parameter can be set to a > 0.8.

comparison among the DSSE degradation characteristic curves produced when the basic scale coefficient is set to 0.1, 0.4, 0.8, and 1.2 is shown in Fig. 11. It is clear from the comparison that when the coefficient is small, the overall degradation curve is distorted, which indicates that the selected parameter is unreasonable. As the parameter value increases, the overall resolution gradually increases and becomes stable, which is consistent with the analytical conclusion derived for the above figure.

### Static symbolization of the loading spectrum

**Figure 12:**
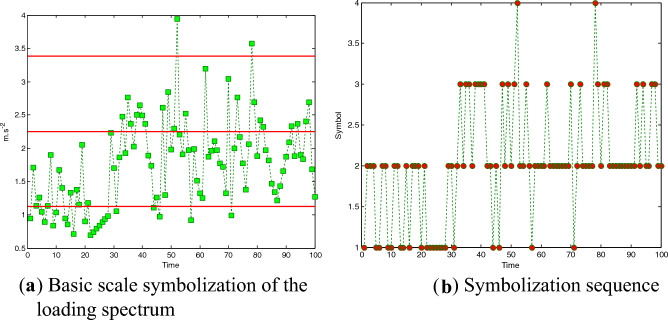
Static divided symbolization. (**a**) Taking a section of the loading spectrum as an example, the static divided symbolization is shown in this figure. (**b**) The amplitude is symbolized according to the basic scale standard, and the symbolization sequence is shown in this figure. Loading spectrum is symbolized as a ‘digitized’ symbol sequence {1,2,3,4}, and the symbol sequence retains the amplitude distribution and variation of the original signal.

First, the process of statically symbolizing the loading spectrum is introduced. The effective value of the first group of vibration loading spectra is 1.8784, and the parameters are set as *a* = 0.6 and *K* = 8. Thus, the basic scale of the divided symbol is *BS*_*0*_ = a*0.666 = 1.1270. Taking a section of the loading spectrum in Fig. 12a as an example, the amplitude is symbolized according to the basic scale standard, and the symbolization sequence is shown in Fig. 12b. Through the static symbolization of the amplitude, the amplitude of the continuous vibration loading spectrum is symbolized as a ‘digitized’ symbol sequence {1,2,3,4}, and the symbol sequence retains the amplitude distribution and variation of the original signal.

### Effectiveness analysis

**Figure 13:**
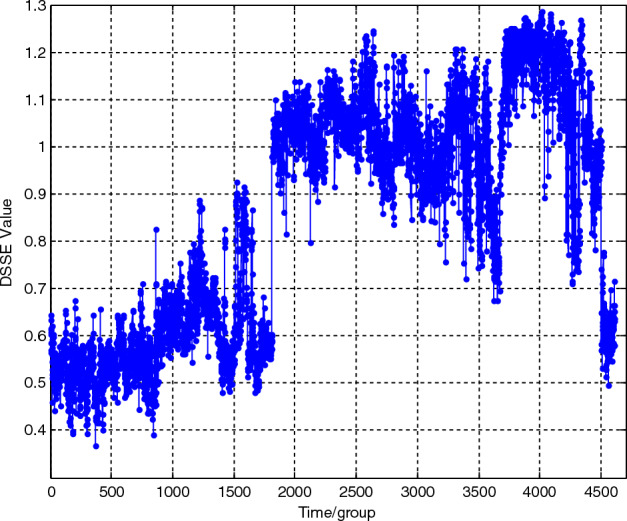
DSSE curve of the lifetime loading spectrum. Feature extraction is performed on each group of vibration loading spectra in turn according to the above parameter settings, and the entropy sequence *dsse*_i_ (*i* = 1,…,4608) is calculated as shown in this figure.

**Figure 14:**
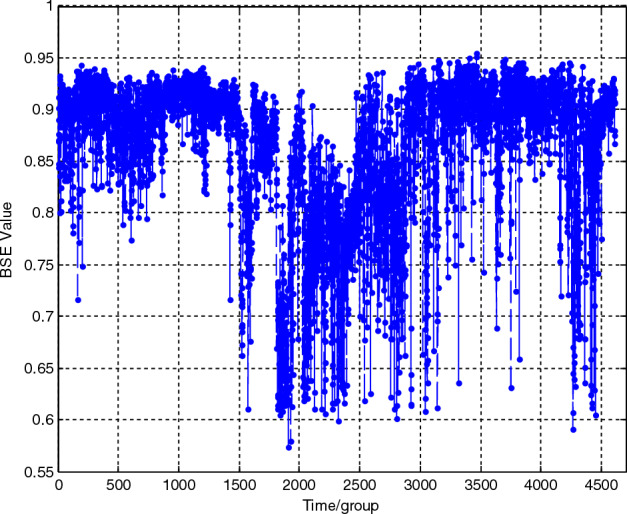
BSE curve of the lifetime loading spectrum. The BSE degradation curve of the lifetime loading spectrum is also obtained and shown in this figure. Through the static symbolization improvement, the DSSE curve exhibits a relatively regular change trend. With the deepening of the performance degradation degree, the value gradually increases, and the degradation phase becomes more obvious.

Feature extraction is performed on each group of vibration loading spectra in turn according to the above parameter settings, and the entropy sequence *dsse*_i_ (*i* = 1,…,4608) is calculated as shown in Fig. 13. For comparison, the BSE parameters are set as *a* = 0.6 and *m* = 4, and the BSE degradation curve of the lifetime loading spectrum is also obtained and shown in Fig. 14.

It is obvious from the comparison that through the static symbolization improvement, the DSSE curve exhibits a relatively regular change trend. With the deepening of the performance degradation degree, the value gradually increases, and the degradation phase becomes more obvious. The main reason for this result is that the unified basic scale *BS*_*0*_ is used in static symbolization. The deeper the degradation degree is, the more large-scale shocks there are, and the more even the symbolized sequence pattern distribution is. Therefore, the information entropy value increases. At the same time, while maintaining the overall increasing trend, the curve is mixed with a certain volatility, which is determined by large noise and many shocks in the vibration signal. In contrast, the BSE curve is less regular and more volatile, making it difficult to accurately evaluate the degradation conditions.

Therefore, the proposed static divided symbol sequence entropy method can reflect the complex evolution principle contained in the signal amplitude during the performance degradation process. The deeper the performance degradation degree is, the larger the value of the degradation feature. Moreover, compared with that of the BSE method, the fluctuation of the curve is greatly reduced, and the overall trend is more obvious, which is more conducive to accurately tracking the performance degradation condition (Supplementary Information).

## Conclusion

To extract the degradation feature of a quay crane hoisting gearbox, a degradation feature extraction method based on static divided symbol sequence entropy is proposed, and the technique is validated with simulations and lifetime vibration signals. The following conclusions are obtained.

1. The proposed static divided symbol sequence entropy technique improves upon the basic scale entropy method, the basic symbolization scale is unified, and the number of symbols and basic scale coefficient are added to more flexibly control the symbolic regions. A verification of the logistic model and the lifetime loading spectrum shows that this technique can be used to sensitively describe the complexity of nonlinear time series. The higher the complexity is, the larger the value; thus, this approach is convenient for tracking the performance degradation process of equipment.
2. The number of symbols and basic scale coefficient are added in this technique, and these two parameters can change the symbol pattern of the symbol sequence, thus affecting the DSSE value. Among them, an increase in the number of symbols gradually increases the information expression ability of the method in the high-amplitude region, improving the complexity resolution of the high-amplitude signal region. In addition, the basic scale coefficient is proposed to control the accuracy of the symbol sequence's ability to express signal information.
3. Compared with three common complexity algorithms, namely, fuzzy entropy, sample entropy, and C0 complexity, the proposed technique has high computational efficiency and strong anti-noise ability while ensuring its degradation feature extraction effect. It can be suitably applied to extract degradation features from mechanical equipment.
4. Limitations of the method. When the initial health status is not clear, its applicability will be limited to a certain extent, which is bound to happen. Because sometimes it is difficult to strictly define or accurately grasp the health state. Therefore, the method proposed in this paper is more inclined to a relative performance degradation evaluation index based on the initial value (current value), rather than an absolute change index. This relativity is specific to the initial value, although sometimes the state is not healthy, but the indicator can also assess the deterioration of the initial state. And this paper only qualitatively analyzes some potential advantages of this method, and does not conduct a detailed quantitative analysis of the noise reduction performance. Our next step is to conduct further research on these aspects.

### Supplementary Information

Supplementary Information.

## Data Availability

The datasets used and analyzed in the current study are available from the corresponding author upon reasonable request.

## Code availability

The code for ‘Simulated Analysis of a Logistic Sequence’ is shared in the Supplementary Information named ‘main.zip’, which includes six ‘.m’ files for the MATLAB platform.
